# Dysregulated metabolism of ceramides and glycosphingolipids in Parkinson’s disease

**DOI:** 10.1016/j.jlr.2025.100955

**Published:** 2025-12-05

**Authors:** Yu-Fong Peng, Szu-Ju Chen, Jeng-Lin Li, Chin-Hsien Lin, Ching-Hua Kuo

**Affiliations:** 1 School of Pharmacy, College of Medicine, National Taiwan University, Taipei, Taiwan; 2 The Metabolomics Core Laboratory, NTU Centers of Genomic and Precision Medicine, National Taiwan University, Taipei, Taiwan; 3 Department of Neurology, National Taiwan University Hospital, Taipei, Taiwan; 4 Department of Neurology, National Taiwan University Hospital Bei-Hu Branch, Taipei, Taiwan; 5 Graduate Institute of Clinical Medicine, College of Medicine, National Taiwan University, Taipei, Taiwan; 6 Ph.D. Program in Translational Medicine, National Taiwan University and Academia Sinica, Taipei, Taiwan; 7 Department of Neurology, National Taiwan University Hospital Jinshan Branch, New Taipei City, Taiwan; 8 Graduate Institute of Biomedical Engineering, College of Medical Science and Technology, National Taiwan University, Taipei, Taiwan; 9 Institute of Molecular Medicine, College of Medicine, National Taiwan University, Taipei, Taiwan; 10 Department of Pharmacy, National Taiwan University Hospital, Taipei, Taiwan

**Keywords:** Parkinson’s disease, sphingolipids, glycosphingolipids, ceramides, glucosylceramides, lactosylceramides, globotriaosylceramides, monosialodihexosylganglioside, *GBA1*, *GLA*

## Abstract

Alterations in sphingolipid metabolism have been implicated in the pathogenesis of Parkinson's disease (PD), yet findings regarding peripheral sphingolipid changes remain inconsistent. This study aimed to elucidate the metabolic profiles of plasma ceramides and glycosphingolipids (GSLs) in patients with PD. We recruited 250 patients with PD and 250 age- and sex-matched neurologically healthy controls. Plasma ceramide and GSL species were quantified using liquid chromatography‒tandem mass spectrometry, complemented by a meta-analysis of the gene expression levels of relevant enzymes in the substantia nigra obtained from Gene Expression Omnibus. A total of 119 sphingolipids were analyzed. Significant differences in plasma sphingolipid species were observed, including increased GSLs and decreased dihydroceramides. Incorporation of 35 significantly altered sphingolipid species enabled discrimination of patients with PD from controls with an AUC of 0.80 (*P* < 0.0001). Notable alterations in lipid ratios were detected, with increases in the monohexosylceramide-to-ceramide ratio as well as the monosialodihexosylganglioside-to-dihexosylceramide and trihexosylceramide-to-dihexosylceramide ratios. We also observed a higher ceramide-to-dihydroceramide ratio and shifts in ceramide characteristics, reflecting changes in the ceramide synthesis pathway. Supporting these findings, meta-analysis revealed changes in the expression of relevant enzymes, including decreased expression of lysosomal hydrolases, such as β-glucocerebrosidase and α-galactosidase, reinforcing the impaired GSL degradation and alteration in ceramide synthesis observed in PD. Our results suggest that altered peripheral ceramide and GSL profiles can discriminate PD from controls. Moreover, we highlight disrupted GSL and ceramide metabolism in PD patients, emphasizing the need for further research to explore the implications of these metabolic disturbances in PD pathogenesis.

## Graphical Abstract

**Figure:**
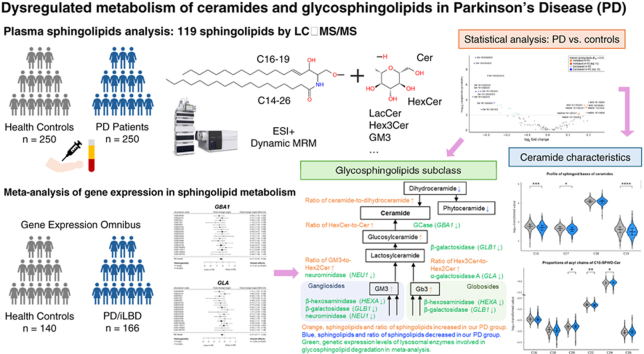

Parkinson’s disease (PD) is the second most common neurodegenerative disease and is characterized by progressive motor impairment and a variety of nonmotor symptoms that markedly reduce patients’ quality of life and contribute to substantial morbidity and mortality (1). PD is thought to result from a complex interplay of aging, genetic mutations, and environmental factors (1). At the molecular level, processes such as protein accumulation, mitochondrial dysfunction, endosomal–lysosomal impairment, and both neuronal and systemic inflammation contribute to disease pathophysiology, which remains incompletely understood (1). The hallmark pathology of PD includes the degeneration of dopaminergic neurons in the substantia nigra (SN) and the deposition of Lewy bodies, which are primarily formed by abnormal α-synuclein protein aggregation (1). Recent evidence has suggested that sphingolipids are key components of the lipid membranes and organelles found within Lewy body cores (2), indicating that disrupted sphingolipid metabolism and glycosphingolipid accumulation may play critical roles in PD pathogenesis (3).

Sphingolipids are major components of cell membranes and regulate cellular metabolism and signal transduction (4). Among them, ceramide is a central bioactive lipid that regulates essential cellular functions, including mitochondrial dynamics and autophagy, and also serves as the precursor to all major sphingolipids (4). Notably, untargeted lipidomics studies have reported significant alterations in ceramides and other sphingolipids in the cerebrospinal fluid (CSF) and plasma of PD patients, enabling their differentiation from healthy individuals (5). However, findings on peripheral ceramide levels have been inconsistent, likely due to variations in sample sizes and the specific sphingolipid isoforms analyzed (6, 7, 8, 9, 10, 11, 12, 13, 14). Moreover, an increasing number of genetic variants linked to glycosphingolipid (GSL) metabolism have been associated with PD risk (15, 16). Among these genes, *GBA1*, which encodes the lysosomal enzyme glucocerebrosidase (GCase) that catabolizes glucosylceramide (GlcCer) into ceramide, is recognized as the most common genetic risk factor for PD (16, 17). Consistently, reduced activity of GCase and other lysosomal hydrolases involved in GSL catabolism has also been observed in patients with PD (7, 18). Despite this genetic and enzymatic evidence, studies quantifying plasma GSL levels have likewise yielded inconsistent results (6, 7, 8, 9, 10, 11, 12, 13, 14, 19).

As therapeutic strategies targeting GSL metabolism are currently under development (20), a detailed characterization of substrate alterations in PD is essential for identifying potential biomarkers and gaining deeper insight into disease pathophysiology. To this end, we conducted a comprehensive targeted lipidomic analysis using liquid chromatography‒tandem mass spectrometry (LC‒MS/MS) to quantify the plasma concentrations of a wide spectrum of GSL and ceramide species in a large cohort of patients with PD and healthy controls, complemented by a meta-analysis of gene expression data for enzymes involved in sphingolipid metabolism to elucidate their pathological relevance and identify candidate biomarkers.

## Materials and methods

### Participants and clinical investigations

PD patients and healthy controls were enrolled from the Movement Disorder Clinic at National Taiwan University Hospital (NTUH), a tertiary referral center in Taiwan. The diagnosis of PD was established according to the UK PD Society Brain Bank Clinical Diagnostic Criteria (21). Healthy controls included neurologically normal individuals accompanying patients, spouse controls, or caregivers sharing similar dietary habits and living environments. Exclusion criteria included vegetarian diet; severe hepatic or renal diseases; irritable bowel syndrome, inflammatory bowel disease, or colon cancer; and use of antibiotics or probiotics within 3 months prior to enrollment. Motor symptom severity in PD patients was assessed during the “off” stage using Hoehn and Yahr (H-Y) staging (22) and the Movement Disorder Society–Unified Parkinson’s Disease Rating Scale (MDS-UPDRS) part III (23). Cognitive performance was assessed during the “on” stage using the Mini-Mental State Examination (MMSE) (24). The study was approved by the institutional ethics board at NTUH, and adhered to the ethical principles outlined in the World Medical Association’s Declaration of Helsinki, with written informed consent obtained from all participants.

### Sample preparation and plasma sphingolipid analysis by LC‒MS/MS

Fasting blood samples were collected in the morning after at least 10 h of fasting. The samples were centrifuged to separate plasma and stored at −80°C until further analysis. For sphingolipid quantification, 20 μl of plasma was extracted with 200 μl of methanol containing internal standards and 0.01% butylated hydroxytoluene as an antioxidant, using a Geno/Grinder 2010 (1000 rpm, 5 min). The internal standards included SphingoSPLASH™ I (catalog no. 330734, Avanti Polar Lipids) and C18:0 GM3-d5 (catalog no. 860073, Avanti Polar Lipids), each at 10 nM. Following centrifugation at 18,000 rcf for 10 min, the supernatants were transferred to new microtubes and stored at −20°C until analyzed by LC‒MS. A pooled QC sample was prepared by combining 1 μl of each extract. To evaluate the association of MS signal and sphingolipid levels, pooled QC samples were serially diluted in methanol at dilution factors of 1, 2, 5, 10, 20, 50, 100, 200, 500, 1000, 2000, 5000, and 10,000.

The LC‒MS system was an Agilent 1290 UHPLC system coupled with an Agilent 6495c triple quadrupole system (Agilent Technologies). An Avantor® ACE® C18-PFP column (2.1 × 100 mm, 1.7 μm, 100 Å, Avantor) was used for the separation. The mobile phase consisted of 60% (v/v) acetonitrile containing 2 mM ammonium acetate (solvent A) and acetonitrile-isopropanol mixtures (3:1, v/v) containing 2 mM ammonium acetate (solvent B). The flow rate was 0.35 ml min^−1^. The gradient profile started with 0% B for 1 min, changed to 50% B in 2 min, changed to 90% B in 6 min, increased to 100% B in 1 min, and was maintained for 1 min. Finally, the column was re-equilibrated to 0% B for 2 min until the next injection. The temperature of the sample reservoir was maintained at 4°C, and the column oven was set at 55°C. The injection volume was 2 μl. Positive electrospray ionization mode was utilized with the following parameters: 250°C dry gas temperature, 15 L min^−1^ dry gas flow rate, 25 psi nebulizer pressure, 300°C sheath gas temperature, 11 L min^−1^ sheath gas flow rate, 3500 V capillary voltage, and 700 V nozzle voltage. The high-pressure and low-pressure RF of the ion funnel were 150 V and 100 V. The MS acquisition was executed in the dynamic multiple reaction monitoring mode containing 200 transitions. The pooled QC samples were analyzed for every 10 study samples.

For data processing of experimental samples, chromatographic peak lists were generated by Agilent MassHunter Qualitative Analysis 10.0 (Agilent Technologies). The exported peak lists included the retention time and peak area for each detected feature. These data were subsequently processed using SphingolipidsID.jl, which annotated peaks by matching precursor–product ion pairs and retention times to entries in the established sphingolipid library (see section ***Plasma sphingolipid identification*** in Supplemental Material) (https://github.com/yufongpeng/SphingolipidsID.jl.2024).

Concentrations of target sphingolipids were calculated by internal standards of the same or most similar lipid polar head class (Supplemental Table S1). Sphingolipid quantification was according to Lipidomics Standard Initiative guidelines (https://lipidomics-standards-initiative.org/); ceramide with sphingosine or sphingadiene (Cer), dihydroceramide (DHS-Cer), monohexosylceramide (HexCer),.dihexosylceramide (Hex2Cer), and monosialodihexosylganglioside (GM3) were quantified at level 2; trihexosylceramides (Hex3Cer) and other lipids were quantified at level 3. TIGERr, the R implementation of TIGER, was then utilized for both intra- and inter-batch technical variation removal (25). The correction process is guided by pooled QC samples, taking into account factors including injection order. After the correction, SphingolipidsID.jl filtered the data with QC samples. Sphingolipids with a coefficient of variation (CV) < 25% across pooled QC injections and a strong MS signal-concentration correlation in serially diluted QC samples (*R*^*2*^ > 0.8) were accepted for statistical analysis.

### Statistical analysis of clinical parameters and plasma sphingolipid levels

Continuous variables are presented as the means ± standard deviations, and categorical variables are expressed as counts (percentages). We examined the homogeneity of variance with the Levene test. Variance homogeneity was assessed with Levene’s test. Between-group comparisons of clinical variables were conducted using Student’s *t* test or ANOVA for normally distributed variables, and Mann–Whitney *U* or Fisher’s exact test otherwise. Sphingolipid concentrations were log-transformed to approximate normal distributions. Group differences were evaluated using multiple *t*-tests, with the Benjamini–Hochberg procedure applied to control the false discovery rate. Diagnostic performance was assessed using the area under the receiver operating characteristic curve (AUC). Associations between sphingolipid levels and disease severity were examined using multivariate linear regression. Lipid set enrichment analysis (LSEA) was performed by LipidSig 2.0 (26). Significantly altered lipids were ranked by log_2_-fold change (log_2_FC), and visualized using Plots.jl (27). Other statistical analyses were conducted with Prism 9 (GraphPad Software, La Jolla, CA, USA), and visualized using Plots.

### Meta-analysis of gene expression in sphingolipid metabolism

We systemically searched the Gene Expression Omnibus (GEO) using Boolean expression keywords (Parkinson's disease) AND “*Homo sapiens*” [porgn:__txid9606] AND (substantia nigra) to derive the complete datasets (28). Eligible datasets met two criteria: (1) experiments were performed on substantia nigra tissue or laser-captured dopaminergic neurons from PD (or incidental Lewy body disease [iLBD]) and controls, and (2) results were analyzed using GEO2R. Datasets not meeting both criteria were excluded. Searches were conducted up to September 2024.

## Results

### Characteristics of the study groups

A total of 500 participants were included in our study, comprising 250 PD patients (67.4 ± 7.7 years old, 34.4% female) and 250 healthy controls (67.4 ± 7.5 years old, 34.0% female). Basic demographics and clinical information are summarized in Table 1. No significant differences were observed between groups with respect to age, sex, BMI, or prevalence of chronic systemic diseases, including hypertension, diabetes mellitus, cardiovascular disease, and cerebrovascular disease. PD patients exhibited a higher prevalence of constipation and cognitive decline compared with controls. Among patients with PD, the average disease duration was 7.4 ± 2.4 years, and the mean MDS-UPDRS part III score during the “off” stage was 35.0 ± 9.9. All patients with PD were receiving antiparkinsonian medication, with an average levodopa equivalent daily dose (LEDD) of 649 ± 206 mg.

**Table 1:** Clinical characteristics of the study participants

	Controls (n = 250)	PD (n = 250)	*P* Value
Age (years)	67.4 ± 7.5	67.4 ± 7.7	0.95
Female, n (%)	85 (34.0)	86 (34.4)	0.93
BMI (kg/m^2^)	22.6 ± 1.5	22.7 ± 1.5	0.99
Hypertension, n (%)	68 (27.2)	53 (21.2)	0.12
Diabetes mellitus, n (%)	61 (24.4)	49 (19.6)	0.19
Cardiovascular disease, n (%)	40 (16.0)	31 (12.4)	0.25
Cerebrovascular disease, n (%)	32 (12.8)	23 (9.2)	0.19
Disease duration (years)	n/a	7.4 ± 2.4	
Hoehn-and-Yahr stage	n/a	3.2 ± 1.0	
MDS-UPDRS part III	n/a	35.0 ± 9.9	
MMSE	28.7 ± 1.8	26.5 ± 2.7	<0.01^b^
LEDD (mg/day)	n/a	649 ± 206	
Constipation, n (%)	103 (41.2)	130 (52.0)	0.02^a^
Creatinine level (mg/dl)	0.92 ± 0.26	0.92 ± 0.24	0.96

Variables are expressed as mean ± standard deviation or number (percentage).

BMI, body mass index; LEDD, levodopa equivalent daily dose; MDS-UPDRS, Movement Disorder Society Unified PD Rating Scale; MMSE, Mini-Mental State Examination; n/a, not available.

a *P* < 0.05.

b *P* < 0.01.

### Comparison of plasma ceramide and GSL profiles

**Fig. 1:**
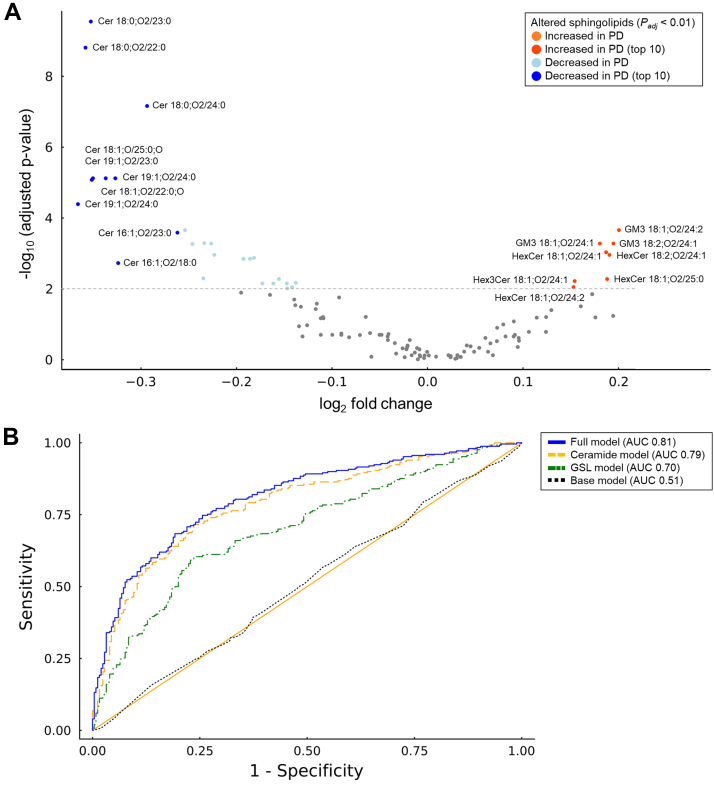
Alterations in plasma levels of ceramides and glycosphingolipids. A total of 119 plasma sphingolipids levels were quantified in 250 patients with Parkinson's disease (PD) and 250 matched healthy controls. A: Log-transformed plasma sphingolipid levels were compared between groups using multiple *t*-tests, with *P*-values adjusted by the Benjamini–Hochberg procedure. Results are shown as a volcano plot, highlighting sphingolipid species significantly elevated (orange) or reduced (light blue) in PD (*P*_*adj*_ < 0.01), with the top 10 species by fold change marked in red (increased) and blue (decreased). B: Receiver–operating characteristic (ROC) analyses assessed the discriminatory performance of these sphingolipids. The area under the ROC curve (AUC) improved from 0.51 using a basic model with age and sex only (Basic model; black dotted line), to 0.70 with glycosphingolipids (GSL model; green dash-dotted line) and 0.79 with ceramides (Ceramide model; orange dash line). The full model involving 35 significantly altered plasma sphingolipid species, alongside age and sex, achieved an AUC of 0.81 (Full model; blue solid line).

To comprehensively assess sphingolipid dysregulation, we quantified 119 ceramide and GSL species with diverse sphingoid bases (SPBs), including sphingosine (SPH), sphigadiene (SPD), dihydrosphingosine (DHS), and phytosphingosine (PHS), and acyl chain compositions (Supplemental Table S1). Species passing quality control were analyzed using multiple *t*-tests (Fig. 1A, Supplemental Table S2). PD patients showed significantly decreased concentrations of ceramides with noncanonical C16-, C17-, and C19-SPHs, ceramides with hydroxylated acyl chains, and DHS-Cer. In contrast, plasma levels of certain GSLs, including HexCer, Hex3Cer, and GM3 containing C18-SPBs and very-long acyl chains, were elevated in PD patients.

We next evaluated the diagnostic performance of significantly altered sphingolipids (*P*_*adj*_ < 0.01) using receiver–operating characteristics (ROC) analysis (Fig. 1B). The AUC improved from 0.51 using a basic model (age and sex only; 95% CI, 0.46–0.56, *P* = 0.745) to 0.70 with GSLs (95% CI 0.65–0.75, *P* < 0.0001) and 0.79 with ceramides (95% CI 0.75–0.83, *P* < 0.0001). The full model, incorporating 35 significantly altered species, achieved an AUC of 0.81 (95% CI 0.77–0.85, *P* < 0.0001), underscoring the diagnostic relevance of sphingolipid alterations in PD.

We then examined the associations between sphingolipid levels and PD disease severity. No significant associations were found between individual lipid species and clinical severity scores (MDS-UPDRS part III, MMSE) after adjusting for confounders, including age, sex, disease duration, and LEDD (Supplemental Table S3).

### Metabolic alterations in GSL subclasses

**Fig. 2:**
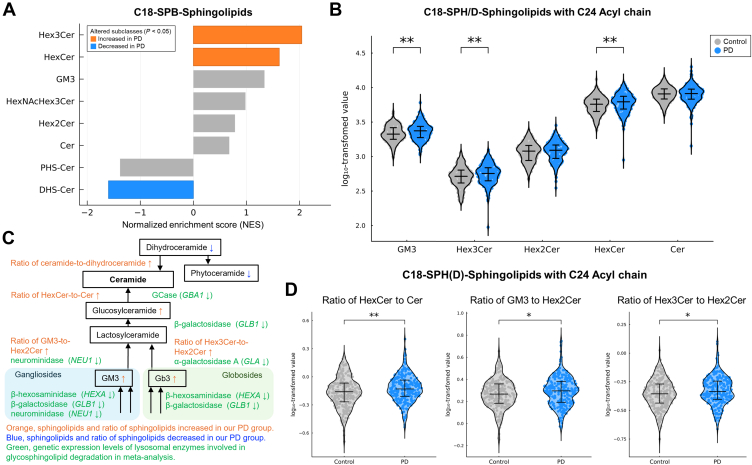
Dysregulation of glycosphingolipids (GSLs) metabolism in patients with Parkinson's disease (PD). GSLs are degraded to ceramides (Cer) by lysosomal enzymes. To illustrate subclass-specific alterations, sphingolipids were categorized by sphingoid base (SPB) and polar head group. A: Lipid set enrichment analysis (LSEA) revealed significant enrichment of sphingolipid subclasses containing C18-SPBs. Trihexosylceramide (Hex3Cer) and monohexosylceramide (HexCer) showed significant increases, whereas dihydroceramide (DHS-Cer) showed a decrease (*P* < 0.05). B: Total concentrations of sphingolipid subclasses with C18-sphingosine or sphingadiene (C18-SPH/D) and C24-acyl chains were log-transformed and compared between patients with PD (blue) and controls (gray) using *t*-tests with Benjamini–Hochberg adjustment. Monosialodihexosylganglioside (GM3), Hex3Cer, and HexCer are significantly increased in PD patients. C: Schematic of the GSL degradation pathway summarizing observed changes in sphingolipid levels and lysosomal enzyme gene expression in PD: orange, sphingolipids and ratio of sphingolipids increased in our PD group; blue, sphingolipids and ratio of sphingolipids decreased in our PD group; green, genetic expression levels of lysosomal enzymes involved in GSL degradation in meta-analysis. D: Ratios of GSL subclasses and ceramides with C18-SPH/D and C24-acyl chains were significantly higher in PD (blue) than in healthy controls (gray). ∗ adjusted *P*-value < 0.05, ∗∗ adjusted *P*-value < 0.01, ∗∗∗ adjusted *P*-value < 0.001, ∗∗∗∗ adjusted *P*-value < 0.0001.

Given dysregulation of the GSL metabolism, we further examined the enrichment trends across ceramide and GSL subclasses. The LSEA of sphingolipid subclasses revealed consistent alterations in GSL metabolism. For subclasses containing canonical C18-SPBs, PD patients exhibited enrichment of HexCer (normalized enrichment score [NES] 1.617; *P* = 0.030), and Hex3Cer (NES 2.041; *P* = 0.003) levels, and depletion of DHS-Cer (NES -1.599; *P* = 0.005) (Fig. 2A).

We then focused on sphingolipids with a C18-SPH or C18-SPD (C18-SPH/D) and a C24-acyl chain, as these species are detected across all subclasses and represent the predominant component. Compared with unaffected controls, patients with PD displayed significantly higher plasma levels of HexCer (6270.1 ± 1862.6 nM vs. 5734.3 ± 1648.5 nM, *P*_*adj*_ = 0.004), Hex3Cer (587.9 ± 189.6 nM vs. 539.2 ± 184.1 nM, *P*_*adj*_ = 0.004), and GM3 (2413.8 ± 705.0 nM vs. 2210.5 ± 592.2 nM, *P*_*adj*_ = 0.004), whereas Hex2Cer and Cer showed no differences (Fig. 2B).

Given the central role of lysosomes in GSL turnover (Fig. 2C) (4), we further examined ratios of GSLs and ceramides. Patients with PD had a significantly elevated HexCer-to-Cer ratio (*P*_*adj*_ = 0.009, Fig. 2D), which remained robust after adjusting for age and sex (odds ratio [OR] = 5.26, 95% CI 1.66–17.19, *P* = 0.005). Moreover, the GM3-to-Hex2Cer (*P*_*adj*_ = 0.030) and Hex3Cer-to-Hex2Cer (*P*_*adj*_ = 0.024) ratios were also significantly elevated in PD patients (Fig. 2D), and these differences persisted after adjustment for age, sex, and HexCer levels (OR for GM3-to-Hex2Cer = 7.14, 95% CI 1.76–29.88, *P* = 0.006; OR for Hex3Cer -to-Hex2Cer = 6.59, 95% CI 1.55–28.96, *P* = 0.012). These shifts suggest impaired lysosomal enzyme activities, such as GCase, neuraminidase, and α-galactosidase, contributing to PD pathogenesis.

### Altered sphingoid base and acyl chain profiles of ceramides

**Fig. 3:**
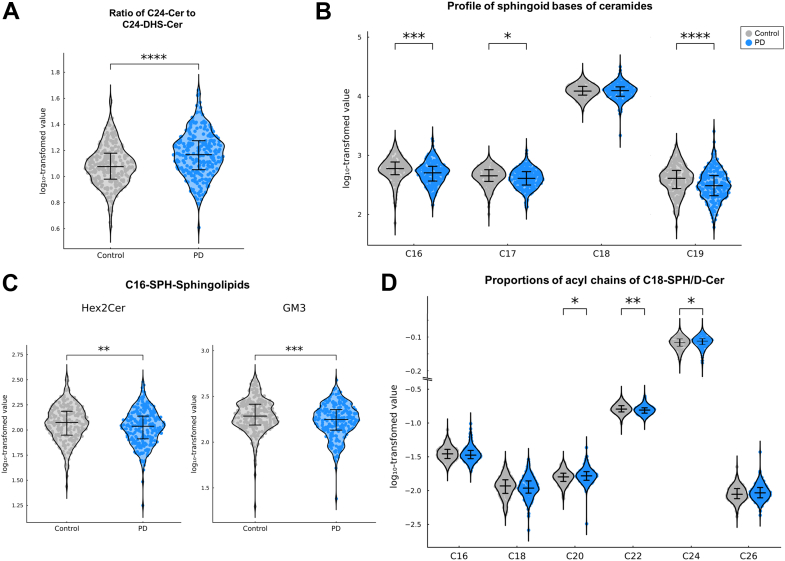
Alterations in ceramides characteristics in patients with Parkinson's disease (PD). Significant changes in ceramide synthesis and metabolism were observed in PD patients (blue markers) compared to healthy controls (gray markers) using *t*-tests, with *P*-values adjusted by the Benjamini–Hochberg procedure. A: The ratio of C24-ceramide (C24-Cer) to C24-dihydroceramide (C24-DHS-Cer) was reduced in patients with PD. B: The profile of ceramide sphingoid bases was assessed by summing species with specific sphingosine (SPH) or sphingadiene (SPD). Log-transformed total concentrations revealed significant reductions in ceramides containing C16-, C17-, and C19-SPHs in PD patients. C: Glycosphingolipids derived from C16-SPH, including dihexosylceramide (Hex2Cer) and monosialodihexosylganglioside (GM3), were also significantly reduced in patients with PD. D: For ceramides with C18-SPH/D and even-numbered acyl chains, the relative distribution of acyl chain lengths showed distinct remodeling: Proportions of C20- and C24-acyl chains were significantly increased, whereas C22 was decreased in patients with PD. ∗ adjusted *P*-value < 0.05, ∗∗ adjusted *P*-value < 0.01, ∗∗∗ adjusted *P*-value < 0.001, and ∗∗∗∗ adjusted *P*-value < 0.0001.

Profiling results indicated perturbations in ceramide metabolism and differential regulation of individual ceramides with different SPBs and acyl chains. To further characterize these alterations, we assessed ceramide structural composition. First, we compared ceramides with dihydroceramides, and found that the C24-Cer-to-C24-DHS-Cer ratio was significantly elevated in PD patients (*P*_*adj*_ < 0.0001; Fig. 3A), indicating increase in ceramide synthesis. We next compared summed concentrations of ceramides categorized by SPBs, and observed significantly decreased levels of noncanonical SPBs, including C16 (*P*_*adj*_ = 0.0013), C17 (*P*_*adj*_ = 0.011), and C19 (*P*_*adj*_ < 0.0001), in PD patients (Fig. 3B). Consistent with this finding, GSLs with C16-SPHs, including Hex2Cer (*P* = 0.009) and GM3 (*P* < 0.001), were also reduced (Fig. 3C). Moreover, for canonical C18-SPH/D, PD patients showed redistribution of acyl chains, with increased proportions of C20 (*P*_*adj*_ = 0.038) and C24 species (*P*_*adj*_ = 0.018) accompanied by reduced C22 species (*P*_*adj*_ = 0.003; Fig. 3D).

### Altered gene expression levels in sphingolipid metabolism

**Fig. 4:**
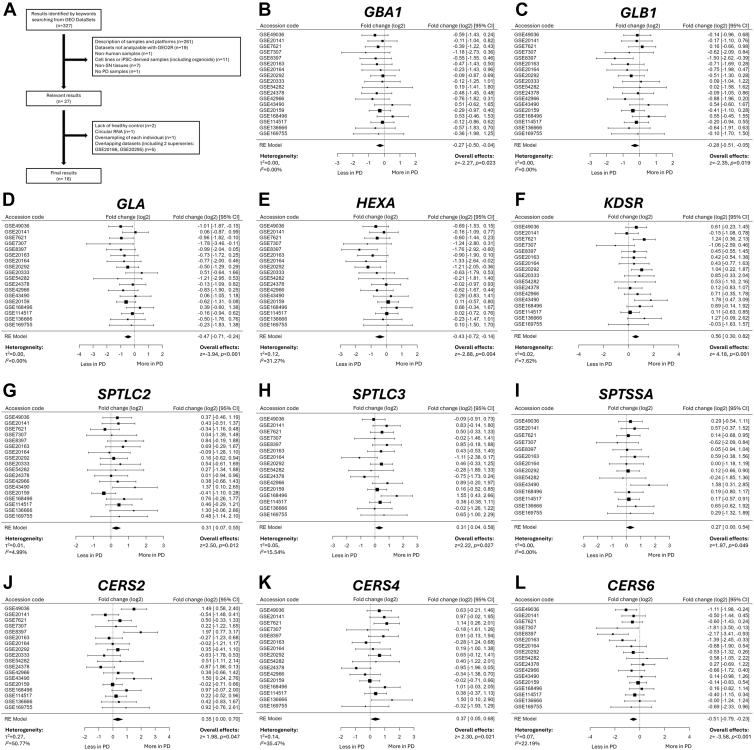
Expression levels of enzymes involved in glycosphingolipid degradation and ceramide synthesis. A GEO search with keywords (Parkinson's disease) AND “Homo sapiens” [porgn:__txid9606] AND (substantia nigra) yielded 327 results, of which 18 met inclusion criteria, encompassing 166 PD/iLBD, and 140 controls. Meta-analysis reveals reduced expression levels of lysosomal enzymes and elevated expression levels of enzymes in de novo ceramide synthesis pathway. A: Meta-analysis workflow. Changes in the mRNA expression of (B) *GBA1*, (C) *GLB1*, (D) *GLA*, (E) *HEXA*, (F) *KDSR*, (G) *SPTLC2*, (H) *SPTLC3*, (I) *SPTSSA*, (J) *CERS2*, (K) *CERS4*, and (L) *CERS6* are observed in the PD/iLBD group.

A GEO search with keywords yielded 327 results, of which 18 met inclusion criteria, encompassing 166 PD/iLBD and 140 controls (Fig. 4A). Meta-analysis of genes related to target enzymes was conducted usingw these datasets, including enzymes responsible for the lysosomal degradation of lipids (*GBA1*, *GLB1*, *GLA*, *NEU1, NEU3, NEU4, HEXA, HEXB,* and *GM2A*) and ceramide synthesis (*SPTLC1, SPTLC2, SPTLC3, KDSR, CERS1*, *CERS2*, *CERS4, CERS5*, *CERS6*, *DEGS1, DEGS2,* and *FADS3*) (Fig. 4, Supplemental Fig. S1).

We found that in SN samples of patients with PD, the expression of *GBA1* (log_2_FC −0.27; 95% CI −0.50 to −0.04, *P* = 0.023; Fig. 4B), *GLB1* (log_2_FC −0.28; 95% CI −0.71 to −0.15, *P* = 0.019; Fig. 4C), *GLA* (log_2_FC −0.47; 95% CI −0.71 to −0.24, *P* < 0.001; Fig. 4D), *HEXA* (log_2_FC −0.43; 95% CI −0.72 to −0.14, *P* = 0.004; Fig. 4E), and *CERS6* (log_2_FC −0.51; 95% CI v0.79 to −0.23, *P* < 0.001; Fig. 4L) was significantly reduced. For *NEU1*, a trend toward decreased expression in patients with PD was also observed (log_2_FC −0.21; 95% CI −0.44 to 0.02, *P* = 0.073; Fig. S1A). In contrast, significantly increased expression levels of *KDSR* (log_2_FC 0.56; 95% CI 0.30–0.82, *P* < 0.001; Fig. 4F), *SPTLC2* (log_2_FC 0.31; 95% CI 0.07–0.55, *P* = 0.012; Fig. 4G)*, SPTLC3* (log_2_FC 0.31; 95% CI 0.04–0.58, *P* = 0.027; Fig. 4H), *SPTSSA* (log_2_FC 0.27; 95% CI 0.00–0.54, *P* = 0.049; Fig. 4I), *CERS2* (log_2_FC 0.35; 95% CI 0.00–0.70, *P* = 0.047; Fig. 4J), and *CERS4* (log_2_FC 0.37; 95% CI 0.05–0.68, *P* = 0.021; Fig. 4K) were observed in SN samples of PD patients. These results align with the plasma lipidomic findings, supporting a model in which PD is characterized by impaired ceramide salvage/lysosomal degradation alongside compensatory activation of de novo synthesis.

## Discussion

Perturbed sphingolipid metabolism has been implicated in PD pathogenesis (29). However, plasma sphingolipids studies have yielded contradictory results, highlighting the need for further validation with larger cohorts (5, 6, 7, 8). Our study, the largest cohort to date, provides a comprehensive plasma sphingolipid profile in well-matched PD patients and controls. We observed significantly elevated plasma GSLs alongside decreased levels of precursor dihydroceramides. Altered GSL-to-ceramide ratios and reduced expression of lysosomal hydrolase genes point toward impaired lysosomal degradation and substrate accumulation in PD. Moreover, compositional shifts in SPBs and acyl chain characteristics suggest additional effects on sphingolipid function. Importantly, these altered sphingolipids robustly discriminated PD patients from controls.

This study covered 119 GSL and ceramide species with diverse headgroups, SPBs, and acyl chains, which is a larger number than reported in most previous PD studies. However, the coverage is lower than in several recent reports (19, 30, 31). Vo *et al.* recently profiled 376 serum GSL species using a four-dimensional reversed-phase LC‒MS workflow (19). They were able to detect more sialylated GSL in human serum, involving sialylated neolacto-series species, tetrasialo gangliosides, and O-acetylated gangliosides with diverse acyl chains and SPBs. Their workflow included multiple purification and fractionation steps, which enabled the detection of low-abundance lipids. In contrast, we employed a simplified extraction protocol and a shorter LC method to achieve higher throughput, allowing the analysis of 250 PD patients and 250 controls (32), though the levels of more complex GSLs beyond GM3, Hex3Cer and HexNAcHex3Cer were below the detection range of our approach. Despite the difference in coverage, both studies exhibited similar increasing trends for Hex3Cer and GM3; however, they found no changes for HexCer, and an increase for Hex2Cer and HexNAcHex3Cer. They also found differential dysregulation of gangliosides for females and males.

Lysosomal dysfunction is recognized as a key factor in PD pathogenesis (33). GCase deficiency, in particular, has been repeatedly demonstrated in brain tissue with Lewy pathology, as well as in CSF and blood from both *GBA1* carriers and sporadic PD patients (7, 34, 35, 36, 37). A moderate inverse association between plasma GlcCer levels and GCase activity has also been documented (37). In our cohort, plasma HexCer, largely reflecting GlcCer (38, 39), was significantly elevated. Considering that C24-Cer comprise over 60% of circulating ceramides, the increase in C24-GlcCer likely contributed substantially to this elevation. Our findings align with recent reports of increased GlcCer in PD plasma across diverse populations (13). By contrast, prior negative studies may have been confounded by small sample sizes or the use of total rather than species-specific measurements (14, 19, 40).

The pathophysiological role of GlcCer in PD remains under investigation (29). In vitro and animal studies have shown that accumulated GlcCer can self-assemble into amyloid-like aggregates, stabilizing toxic α-synuclein oligomers and promoting α-synuclein aggregation (41, 42). In turn, α-synuclein oligomers interfere with the intracellular trafficking of GCase, leading to reduced lysosomal GCase activity and further accumulation of GlcCer, creating a vicious cycle (42). Human studies reinforce this mechanism, with GlcCer levels correlating with α-synuclein pathology and peripheral neuropathies (43). Given that GlcCer is the precursor of higher-order glycosphingolipids, its accumulation may also dysregulate upstream globoside and ganglioside metabolism in the salvage pathway of ceramide synthesis (Fig. 2C) (44). Moreover, reduced activities of related lysosomal enzymes in the central nervous system (CNS) have been previously reported in PD patients (7, 45).

Among these substrates, GM3, the simplest gangliosides, was consistently reported as increased in both the nervous system and plasma of PD patients (7, 8, 19, 37); however, its role in PD pathogenesis remained unclear. In a lipopolysaccharide (LPS)-induced PD model, C18-GM3 was shown to engage the TLR4-driven inflammatory cascade, ultimately protecting dopaminergic terminals and improving motor deficits (46). Synthetic C18-GM3 derivatives also conferred neuroprotection in SH-SY5Y cells and promoted neurite outgrowth in primary neurons (47). Furthermore, studies using in vitro lipid vesicles and monolayers demonstrated that higher content of GM3, especially very-long acyl chain GM3 such as C24-GM3, increased α-synuclein-membrane lipids interactions, potentially triggering α-synuclein membrane insertion and PD pathology (48, 49). Notably, our study revealed elevated plasma C24-GM3 and higher C24-GM3-to-Hex2Cer ratios in PD patients, while C18-GM3 remained unchanged. These results highlight the potential variation in GM3 metabolism for different acyl chain lengths and their functional difference in PD pathogenesis.

Globosides, including globotriaosylceramide (Gb3), have received less attention in PD. Although α-galactosidase deficiency has been reported in both CNS and peripheral tissues, prior studies reported inconsistent Gb3 alterations (7, 18, 19, 45, 50). Here, we observed increased plasma C24-Hex3Cer, mainly corresponding to C24-Gb3 (51, 52), and decreased *GLA* expression in PD substantia nigra, suggesting a role for impaired Gb3 turnover. Mechanistically, Gb3 exhibits strong affinity for α-synuclein and can activate toll-like receptor 4 (TLR4)-mediated proinflammatory signaling (49, 53), both of which may contribute to disease progression.

Because the salvage pathway contributes over half of ceramide synthesis, its impairment may drive compensatory upregulation of the de novo synthesis pathway (4, 54). Supporting this, our meta-analysis showed increased expression of serine palmitoyltransferase (SPT) subunits (*SPTLC2*, *SPTLC3*, *SPTSSA*) and *KDSR* in PD substantia nigra. Plasma lipidomics revealed decreased dihydroceramides and an increased SPH/D-Cer-to-DHS-Cer ratio, consistent with enhanced conversion to maintain ceramide homeostasis.

SPB–specific changes further highlight selective regulation. We detected reductions in the levels of the C16-SPB, C17-SPB, and C19-SPB ceramides, but no alteration for C18-SPB ceramides. SPB length is largely dictated by the SPT complex (4). The SPTLC1-SPTLC2-SPTSSA complex preferentially uses C16-CoA, generating C18-SPB sphingolipids (55). Our meta-analysis revealed upregulated genetic expression of *SPTLC2* and *SPTSSA*, consistent with stable C18-SPB levels. In contrast, although *SPTLC3* expression was also upregulated, downstream ceramide synthase activity for precursor C16-DHS appeared insufficient, leading to reduced C16-SPB sphingolipids (56). Other noncanonical SPB sphingolipids may be further limited by unchanged *SPTLC1* and *SPTSSB* expression. These mechanisms likely explain the reduced noncanonical SPB sphingolipids observed. Until now, very few studies have addressed functional roles of these noncanonical SPBs, warranting further investigation.

Acyl chain remodeling also emerged as a key feature. PD patients exhibited higher proportions of C20- and C24-ceramides and reduced C22 species. Ceramide synthases, a family of six enzymes, dictate acyl chain incorporation (57). C20-acyl chains are primarily utilized by ceramide synthase 4 (CerS4) (57). Conversely, ceramide synthase 2 (CerS2) favors C22- to C24-acyl chains (57). Our meta-analysis revealed higher expression of *CERS2* and *CERS4*, as well as reduced *CERS5*. Notably, the activity of ceramide synthases is modulated by dimerization (57). The increased expression of *CERS4* and *CERS2* may lead to the formation of heterodimers, boosting the production of C24:1- and C20-ceramides while reducing other chain lengths (57). Animal models further demonstrate that excess very-long acyl chain ceramides, particularly those generated by CerS2, promote oxidative stress, mitochondrial impairments, mitophagy, and apoptosis (58).

Through our sphingolipidomics analysis, we observed a general elevation of GSLs, an increased ceramide-to-dihydroceramide ratio and shifts in ceramide characteristics, suggesting that PD may involve a general defect in sphingolipid catabolism. Indeed, the interaction between α-synuclein and lipid metabolism has long been recognized (59). Recent studies using human induced pluripotent stem cell-derived neurons and neuronal cell models have shown that α-synuclein pathology induces alterations in lipid composition, including sphingolipids (59, 60). In addition, these lipid changes are most prominent in the endoplasmic reticulum (ER) and mitochondria-associated ER membrane domains, which are identified as the subcellular regions where key pathophysiological events likely occur, though the precise mechanisms remain to be elucidated (59). Future investigations are warranted to clarify the mechanistic basis of sphingolipid dysregulation in PD and to determine its role in disease initiation and progression.

Our study has several limitations. First, this study did not distinguish major GSL species from other possible isomers. Although the observed HexCer and Hex3Cer elevations likely reflect increases in GlcCer and Gb3, contributions from other isomers cannot be excluded. Second, our analyses focused exclusively on sphingolipids. Other lysosome-associated lipids, such as lysobisphosphatidic acid, should be incorporated in future studies to provide a broader assessment of lipid dysregulation and lysosomal dysfunction in PD. Third, unmeasured factors such as diet and lipid metabolism may influence circulating sphingolipids, though our tightly matched control cohort likely minimized these confounders with comparable BMI, age of the participants, and prevalence of chronic systemic diseases. Additionally, our control cohort primarily comprised close associates or caregivers of patients, who likely shared similar dietary habits. Fourth, we did not examine the influence of medications other than anti-PD drugs on sphingolipid levels. Although no evidence currently links common medications to peripheral sphingolipid dysregulation, our large sample size and the use of lipid ratio analyses likely mitigated potential drug-related bias. Fifth, our study did not assess genetic heterogeneity and the activities of enzymes related to sphingolipid metabolism. Although the plasma sphingolipidomics data (primarily from East Asian population) and the post-mortem SN transcriptomics data (mainly from Western populations) show consistent trends, it remains difficult to infer a causal relationship. Future multicenter studies involving diverse ethnic populations that concurrently examine sphingolipid concentrations and enzyme activities are needed to validate their roles in PD pathogenesis.

In conclusion, our findings demonstrate distinct plasma GSL and ceramide alterations that discriminate PD patients from controls. Previous studies have mainly focused on HexCer. Through our comprehensive profiling of sphingolipids, we observed upregulated GM3 and Gb3, as well as decreased levels of noncanonical SPBs and redistribution of acyl chains. We propose that lysosomal hydrolase deficiencies, including GCase and α-galactosidase, and compensatory shifts in ceramide synthesis underlie these perturbations. These alterations may influence α-synuclein aggregation, inflammation, and mitochondrial health, offering new insights into PD pathophysiology and potential therapeutic targets.

## Data Availability

Sphingolipids concentration data are provided in Supporting Data. Clinical data and meta-analysis data are available from the corresponding author upon request.

## Supplemental Data

This article contains supplemental data (61, 62).

## Conflict of Interest

The authors declare that they do not have any conflicts of interest with the content of this article.
