# Supplementary material for: Dysregulated metabolism of ceramides and glycosphingolipids in Parkinson’s disease
Source: J Lipid Res. 2025 Dec 5;67(1):100955. doi: 10.1016/j.jlr.2025.100955 (PMC12828830; doi:10.1016/j.jlr.2025.100955)
Supplement: Supplemental Material [file mmc1.pdf]

Supplemental Material

Plasma sphingolipid identification

Sphingolipids were identified from healthy human plasma using precursor ion scans targeting multiple characteristic product ions, including sialic acid (NeuAc) and sphingoid base (SPB) fragments in positive ion mode. Precursor ion mode data were first processed by MZmine 3 to generate chromatographic peak lists [1]. A sphingolipid library was then constructed using the Julia package SphingolipidsID.jl [2]. Peaks with a coefficient of variation (CV) greater than 50% were excluded. Lipid identification was performed by matching protonated precursor–SPB ion pairs between the *in-silico* sphingolipid library and experimental data. After extensive ion-pair matching, signals were grouped according to retention time and in-source fragmentation relationships. Within each group, lipids showing the largest signal intensity or molecular mass were designated as potential parent species, while the others were annotated as in-source fragments.

Expected in-source fragment ions were used as class identification qualifiers: for ceramides, a neutral loss of water; for HexCer, a neutral loss of water or a hexose (Hex); for Hex2Cer, a neutral loss of water or two Hex units; for Hex3Cer, a neutral loss of a dehydroxylated Hex, a Hex, or three Hex units; and for GM3, a neutral loss of a dehydroxylated NeuAc-Hex, a NeuAc-Hex, or the entire sugar moiety. Additional SPB fragments were used as chain identification qualifiers.

A retention time regression model was established using class, carbon number, double bonds, and

hydroxylation as predictors, based on commonly detected plasma sphingolipids. Retention time tolerances were set at 1.0 min for HexNAcHex3Cer, 0.7 min for species with more than two hydroxylations, and 0.5 min for all other lipids [3]. Co-eluting isobars were manually inspected according to predicted retention time, class and chain qualifiers, and CV values. Finally, the lipid library was validated using multiple reaction monitoring, and the resulting transition list was used for sample analysis.

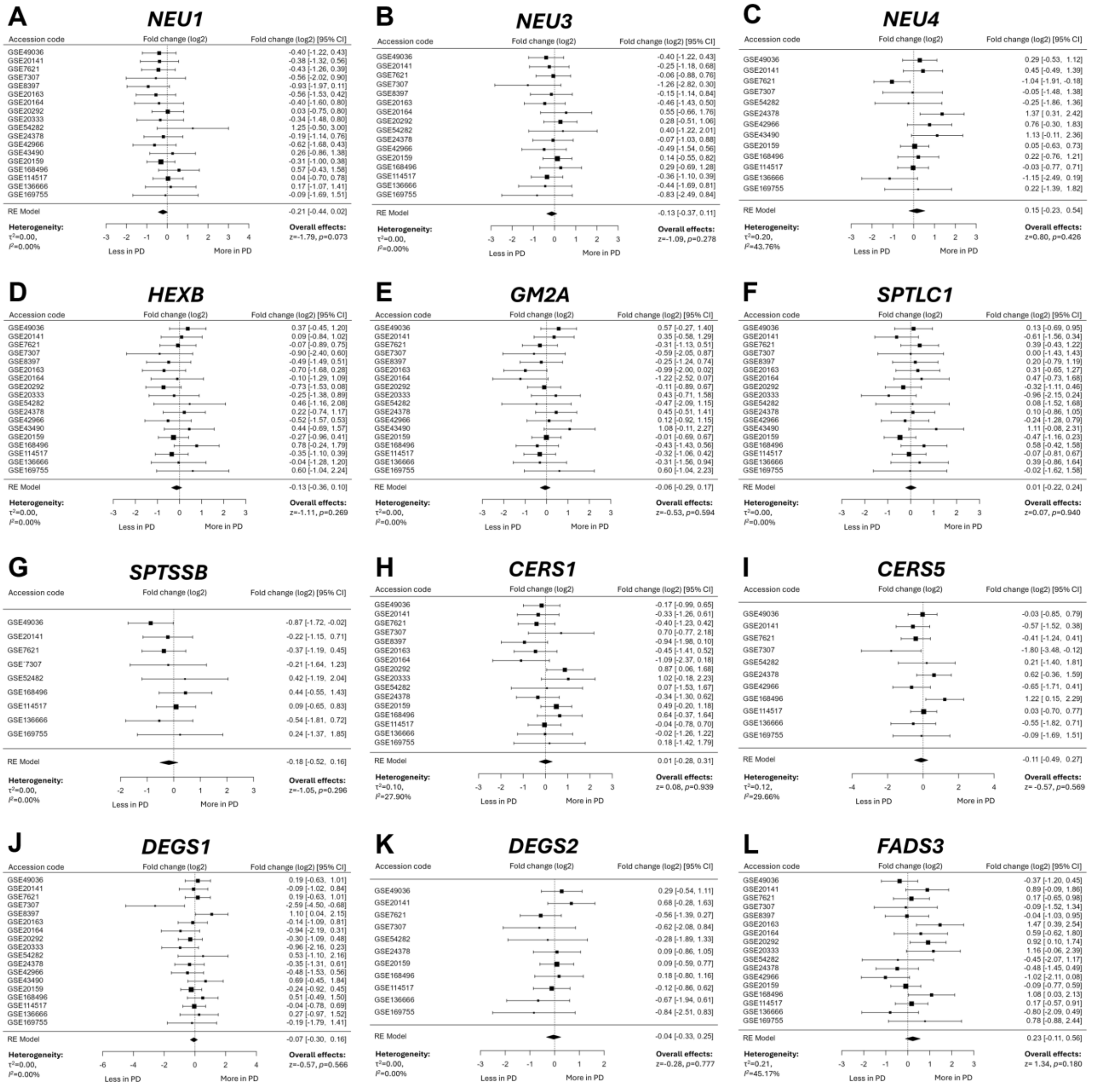

Figure S1. Expression levels of lysosomal enzymes involved in glycosphingolipid degradation and ceramide synthesis. No siginificant changes in the mRNA expression of (A) *NEU1*, (B) *NEU3*, (C) *NEU4*, (D) *HEXB*, (E) *GM2A*, (F) *SPTLC1*, (G) *SPTSSB*, (H) *CERS1*, (I) *CERS5*, (J) *DEGS1*, (K) *DEGS2*, and (L) *FADS3* were observed in the PD/iLBD group.

Table S1. Transition, internal standards, retention time (RT), coefficient of variation (CV) and coefficient of determination (R^2) of targeted sphingolipid species and internal standards in our study.

	Transition	Internal standard	RT (min)	CV	R^2
Cer 18:1;O2[D7]/15:0	531.5→271.3	NA	6.8	NA	1.00
Cer 18:0;O2[D7]/13:0	505.5→273.3	NA	6.4	NA	1.00
GlcCer 18:1;O2[D7]/15:0	693.6→271.3	NA	5.9	NA	1.00
GalCer 18:1;O2[D7]/13:0	665.6→271.3	NA	5.2	NA	1.00
LacCer 18:1;O2[D7]/15:0	855.6→271.3	NA	5.6	NA	1.00
GM3 18:1;O2/18:0[D5]	1186.8→264.3	NA	4.2	NA	0.99
Cer 16:1;O2/16:0	510.5→236.2	Cer 18:1;O2[D7]/15:0	6.5	3.7%	1.00
Cer 16:1;O2/18:0	538.5→236.2	Cer 18:1;O2[D7]/15:0	7.2	8.2%	1.00
Cer 16:1;O2/20:0	566.6→236.2	Cer 18:1;O2[D7]/15:0	7.8	11.8%	1.00
Cer 16:1;O2/22:0	594.6→236.2	Cer 18:1;O2[D7]/15:0	8.4	9.4%	0.99
Cer 16:1;O2/23:0	608.6→236.2	Cer 18:1;O2[D7]/15:0	8.7	14.6%	0.99
Cer 16:1;O2/24:0	622.6→236.2	Cer 18:1;O2[D7]/15:0	8.9	9.1%	0.97
Cer 16:1;O2/24:1	620.6→236.2	Cer 18:1;O2[D7]/15:0	8.3	15.4%	1.00
Cer 16:1;O2/25:0	636.6→236.2	Cer 18:1;O2[D7]/15:0	9.2	11.0%	0.97

Cer 17:1;O2/16:0	524.5→250.3	Cer 18:1;O2[D7]/15:0	6.8	2.8%	0.99
Cer 17:1;O2/22:0	608.6→250.3	Cer 18:1;O2[D7]/15:0	8.6	14.7%	0.97
Cer 17:1;O2/23:0	622.6→250.3	Cer 18:1;O2[D7]/15:0	8.9	9.3%	0.96
Cer 17:1;O2/23:1	620.6→250.3	Cer 18:1;O2[D7]/15:0	8.4	10.1%	1.00
Cer 17:1;O2/24:0	636.6→250.3	Cer 18:1;O2[D7]/15:0	9.1	8.8%	0.95
Cer 17:1;O2/24:1	634.6→250.3	Cer 18:1;O2[D7]/15:0	8.7	10.0%	0.99
Cer 17:1;O2/25:1	648.6→250.3	Cer 18:1;O2[D7]/15:0	8.9	9.3%	0.99
Cer 17:1;O2/25:2	646.6→250.3	Cer 18:1;O2[D7]/15:0	8.3	15.6%	1.00
Cer 18:0;O2/22:0	624.6→284.3	Cer 18:0;O2[D7]/13:0	9.1	14.1%	0.93
Cer 18:0;O2/23:0	638.6→284.3	Cer 18:0;O2[D7]/13:0	9.3	13.5%	0.94
Cer 18:0;O2/24:0	652.7→284.3	Cer 18:0;O2[D7]/13:0	9.5	7.4%	0.94
Cer 18:0;O2/24:1	650.6→284.3	Cer 18:0;O2[D7]/13:0	9.0	14.0%	0.96
Cer 18:0;O3/22:0	640.6→282.3	Cer 18:0;O2[D7]/13:0	8.6	8.6%	0.94
Cer 18:0;O3/23:0	654.6→282.3	Cer 18:0;O2[D7]/13:0	8.8	9.6%	0.95
Cer 18:0;O3/24:0	668.7→282.3	Cer 18:0;O2[D7]/13:0	9.1	8.5%	0.94
Cer 18:0;O3/24:1	666.6→282.3	Cer 18:0;O2[D7]/13:0	8.5	13.9%	0.98
Cer 18:1;O/20:0	578.6→266.3	Cer 18:1;O2[D7]/15:0	8.5	15.8%	1.00
Cer 18:1;O/21:0	592.6→266.3	Cer 18:1;O2[D7]/15:0	8.7	16.4%	0.99

Cer 18:1;O/22:0	606.6→266.3	Cer 18:1;O2[D7]/15:0	9.0	9.7%	1.00
Cer 18:1;O/23:0	620.6→266.3	Cer 18:1;O2[D7]/15:0	9.2	9.6%	0.99
Cer 18:1;O/24:0	634.7→266.3	Cer 18:1;O2[D7]/15:0	9.4	8.8%	0.98
Cer 18:1;O/24:1	632.6→266.3	Cer 18:1;O2[D7]/15:0	8.9	9.8%	1.00
Cer 18:1;O/25:0	648.7→266.3	Cer 18:1;O2[D7]/15:0	9.6	8.1%	0.98
Cer 18:1;O/25:0;O	664.7→266.3	Cer 18:1;O2[D7]/15:0	9.5	8.1%	0.97
Cer 18:1;O2/16:0	538.5→264.3	Cer 18:1;O2[D7]/15:0	7.2	7.4%	0.99
Cer 18:1;O2/18:0	566.6→264.3	Cer 18:1;O2[D7]/15:0	7.8	11.4%	0.98
Cer 18:1;O2/20:0	594.6→264.3	Cer 18:1;O2[D7]/15:0	8.4	14.8%	0.95
Cer 18:1;O2/22:0	622.6→264.3	Cer 18:1;O2[D7]/15:0	8.9	14.5%	0.95
Cer 18:1;O2/22:0;O	638.6→264.3	Cer 18:1;O2[D7]/15:0	8.6	8.7%	0.92
Cer 18:1;O2/22:1	620.6→264.3	Cer 18:1;O2[D7]/15:0	8.3	15.6%	0.97
Cer 18:1;O2/23:0	636.6→264.3	Cer 18:1;O2[D7]/15:0	9.1	8.7%	0.94
Cer 18:1;O2/24:0	650.6→264.3	Cer 18:1;O2[D7]/15:0	9.4	8.0%	0.94
Cer 18:1;O2/24:0;O	666.6→264.3	Cer 18:1;O2[D7]/15:0	9.1	14.5%	0.94
Cer 18:1;O2/24:1	648.6→264.3	Cer 18:1;O2[D7]/15:0	8.8	8.9%	0.97
Cer 18:1;O2/24:1;O	664.6→264.3	Cer 18:1;O2[D7]/15:0	8.5	8.1%	0.94
Cer 18:1;O2/25:0	664.7→264.3	Cer 18:1;O2[D7]/15:0	9.6	7.9%	0.94

Cer 18:1;O2/25:1	662.6→264.3	Cer 18:1;O2[D7]/15:0	9.0	13.8%	0.94
Cer 18:1;O2/26:0	678.7→264.3	Cer 18:1;O2[D7]/15:0	9.8	17.1%	0.93
Cer 18:1;O2/26:1	676.7→264.3	Cer 18:1;O2[D7]/15:0	9.3	11.2%	0.94
Cer 18:2;O2/16:0	536.5→262.3	Cer 18:1;O2[D7]/15:0	6.6	3.9%	1.00
Cer 18:2;O2/18:0	564.5→262.3	Cer 18:1;O2[D7]/15:0	7.2	8.7%	1.00
Cer 18:2;O2/20:0	592.6→262.3	Cer 18:1;O2[D7]/15:0	7.9	16.7%	1.00
Cer 18:2;O2/22:0	620.6→262.3	Cer 18:1;O2[D7]/15:0	8.4	9.0%	1.00
Cer 18:2;O2/23:0	634.6→262.3	Cer 18:1;O2[D7]/15:0	8.7	8.2%	1.00
Cer 18:2;O2/24:0	648.6→262.3	Cer 18:1;O2[D7]/15:0	8.9	8.9%	0.99
Cer 18:2;O2/24:1	646.6→262.3	Cer 18:1;O2[D7]/15:0	8.3	15.2%	1.00
Cer 18:2;O2/25:0	662.6→262.3	Cer 18:1;O2[D7]/15:0	9.2	14.2%	0.99
Cer 18:2;O2/26:0	676.7→262.3	Cer 18:1;O2[D7]/15:0	9.4	9.7%	0.99
Cer 19:1;O2/22:0	636.6→278.3	Cer 18:1;O2[D7]/15:0	9.1	9.1%	0.97
Cer 19:1;O2/23:0	650.6→278.3	Cer 18:1;O2[D7]/15:0	9.3	8.9%	0.97
Cer 19:1;O2/24:0	664.7→278.3	Cer 18:1;O2[D7]/15:0	9.5	7.9%	0.96
Cer 19:1;O2/24:1	662.6→278.3	Cer 18:1;O2[D7]/15:0	9.0	9.1%	1.00
GM3 16:1;O2/20:0	1181.8→236.2	GM3 18:1;O2/18:0[D5]	4.2	3.3%	1.00
GM3 16:1;O2/22:0	1209.8→236.2	GM3 18:1;O2/18:0[D5]	4.6	3.8%	0.99

GM3 18:1;O2/16:0	1153.7→264.3	GM3 18:1;O2/18:0[D5]	3.6	2.9%	1.00
GM3 18:1;O2/18:0	1181.8→264.3	GM3 18:1;O2/18:0[D5]	4.1	2.5%	0.99
GM3 18:1;O2/20:0	1209.8→264.3	GM3 18:1;O2/18:0[D5]	4.6	3.9%	0.98
GM3 18:1;O2/22:0	1237.8→264.3	GM3 18:1;O2/18:0[D5]	5.0	4.3%	0.96
GM3 18:1;O2/22:0;O	1253.8→264.3	GM3 18:1;O2/18:0[D5]	4.9	5.1%	0.96
GM3 18:1;O2/23:0	1251.8→264.3	GM3 18:1;O2/18:0[D5]	5.3	4.3%	0.96
GM3 18:1;O2/24:0	1265.8→264.3	GM3 18:1;O2/18:0[D5]	5.5	5.8%	0.96
GM3 18:1;O2/24:0;O	1281.8→264.3	GM3 18:1;O2/18:0[D5]	5.4	7.1%	0.96
GM3 18:1;O2/24:1	1263.8→264.3	GM3 18:1;O2/18:0[D5]	5.0	4.2%	0.99
GM3 18:1;O2/24:1;O	1279.8→264.3	GM3 18:1;O2/18:0[D5]	4.9	5.0%	0.98
GM3 18:1;O2/24:2	1261.8→264.3	GM3 18:1;O2/18:0[D5]	4.6	3.8%	0.99
GM3 18:2;O2/16:0	1151.7→262.3	GM3 18:1;O2/18:0[D5]	3.0	3.2%	1.00
GM3 18:2;O2/18:0	1179.7→262.3	GM3 18:1;O2/18:0[D5]	3.7	3.2%	1.00
GM3 18:2;O2/22:0	1235.8→262.3	GM3 18:1;O2/18:0[D5]	4.6	3.7%	1.00
GM3 18:2;O2/24:0	1263.8→262.3	GM3 18:1;O2/18:0[D5]	5.1	4.6%	1.00
GM3 18:2;O2/24:1	1261.8→262.3	GM3 18:1;O2/18:0[D5]	4.6	4.0%	1.00
Hex2Cer 16:1;O2/16:0	834.6→236.2	LacCer 18:1;O2[D7]/15:0	5.3	3.7%	1.00
Hex2Cer 18:1;O2/14:0	834.6→264.3	LacCer 18:1;O2[D7]/15:0	5.2	4.2%	1.00

Hex2Cer 18:1;O2/16:0	862.6→264.3	LacCer 18:1;O2[D7]/15:0	6.0	2.9%	0.99
Hex2Cer 18:1;O2/22:0	946.7→264.3	LacCer 18:1;O2[D7]/15:0	7.9	4.6%	0.94
Hex2Cer 18:1;O2/23:0	960.7→264.3	LacCer 18:1;O2[D7]/15:0	8.2	5.5%	0.93
Hex2Cer 18:1;O2/24:0	974.8→264.3	LacCer 18:1;O2[D7]/15:0	8.5	5.5%	0.93
Hex2Cer 18:1;O2/24:1	972.7→264.3	LacCer 18:1;O2[D7]/15:0	7.8	5.2%	0.98
Hex2Cer 18:1;O2/24:2	970.7→264.3	LacCer 18:1;O2[D7]/15:0	7.3	6.2%	1.00
Hex2Cer 18:2;O2/16:0	860.6→262.3	LacCer 18:1;O2[D7]/15:0	5.4	2.7%	1.00
Hex2Cer 18:2;O2/24:0	972.7→262.3	LacCer 18:1;O2[D7]/15:0	8.0	5.6%	0.99
Hex2Cer 18:2;O2/24:1	970.7→262.3	LacCer 18:1;O2[D7]/15:0	7.3	6.0%	1.00
Hex3Cer 18:1;O2/16:0	1024.7→264.3	LacCer 18:1;O2[D7]/15:0	5.7	21.2%	0.99
Hex3Cer 18:1;O2/18:0	1052.7→264.3	LacCer 18:1;O2[D7]/15:0	6.5	4.9%	0.95
Hex3Cer 18:1;O2/22:0	1108.8→264.3	LacCer 18:1;O2[D7]/15:0	7.8	5.9%	0.94
Hex3Cer 18:1;O2/23:0	1122.8→264.3	LacCer 18:1;O2[D7]/15:0	8.1	12.9%	0.93
Hex3Cer 18:1;O2/24:0	1136.8→264.3	LacCer 18:1;O2[D7]/15:0	8.3	4.9%	0.93
Hex3Cer 18:1;O2/24:1	1134.8→264.3	LacCer 18:1;O2[D7]/15:0	7.7	5.9%	0.96
Hex3Cer 18:2;O2/24:0	1134.8→262.3	LacCer 18:1;O2[D7]/15:0	7.8	5.5%	0.99
Hex3Cer 18:2;O2/24:1	1132.8→262.3	LacCer 18:1;O2[D7]/15:0	7.1	6.5%	0.97
HexCer 17:1;O2/24:0	798.7→250.3	GlcCer 18:1;O2[D7]/15:0	8.5	4.9%	0.95

HexCer 18:1;O2/16:0	700.6→264.3	GlcCer 18:1;O2[D7]/15:0	6.3	2.2%	1.00
HexCer 18:1;O2/16:0;O	716.6→264.3	GlcCer 18:1;O2[D7]/15:0	6.1	2.5%	0.98
HexCer 18:1;O2/18:0	728.6→264.3	GlcCer 18:1;O2[D7]/15:0	7.0	4.4%	0.97
HexCer 18:1;O2/20:0	756.6→264.3	GlcCer 18:1;O2[D7]/15:0	7.6	5.0%	0.93
HexCer 18:1;O2/22:0	784.7→264.3	GlcCer 18:1;O2[D7]/15:0	8.2	3.5%	0.95
HexCer 18:1;O2/22:0;O	800.7→264.3	GlcCer 18:1;O2[D7]/15:0	8.0	4.4%	0.94
HexCer 18:1;O2/23:0	798.7→264.3	GlcCer 18:1;O2[D7]/15:0	8.5	3.8%	0.94
HexCer 18:1;O2/23:0;O	814.7→264.3	GlcCer 18:1;O2[D7]/15:0	8.3	4.9%	0.93
HexCer 18:1;O2/24:0	812.7→264.3	GlcCer 18:1;O2[D7]/15:0	8.7	4.1%	0.95
HexCer 18:1;O2/24:0;O	828.7→264.3	GlcCer 18:1;O2[D7]/15:0	8.6	5.0%	0.94
HexCer 18:1;O2/24:1	810.7→264.3	GlcCer 18:1;O2[D7]/15:0	8.1	3.9%	0.99
HexCer 18:1;O2/24:1;O	826.7→264.3	GlcCer 18:1;O2[D7]/15:0	8.0	4.7%	0.96
HexCer 18:1;O2/24:2	808.7→264.3	GlcCer 18:1;O2[D7]/15:0	7.6	13.0%	0.98
HexCer 18:1;O2/25:0	826.7→264.3	GlcCer 18:1;O2[D7]/15:0	8.9	5.5%	0.95
HexCer 18:2;O2/22:0	782.7→262.3	GlcCer 18:1;O2[D7]/15:0	7.7	4.2%	0.99
HexCer 18:2;O2/23:0	796.7→262.3	GlcCer 18:1;O2[D7]/15:0	8.0	12.4%	1.00
HexCer 18:2;O2/24:0	810.7→262.3	GlcCer 18:1;O2[D7]/15:0	8.2	12.3%	1.00
HexCer 18:2;O2/24:1	808.7→262.3	GlcCer 18:1;O2[D7]/15:0	7.6	4.8%	0.99

HexNAcHex3Cer 18:1;O2/16:0	1227.8→264.3	LacCer 18:1;O2[D7]/15:0	5.6	4.3%	0.99
HexNAcHex3Cer 18:1;O2/24:1	1337.9→264.3	LacCer 18:1;O2[D7]/15:0	7.5	7.9%	0.97
HexNAcHex3Cer 18:2;O2/16:0	1225.7→262.3	LacCer 18:1;O2[D7]/15:0	4.9	12.7%	0.96

Table S2. Sphingolipids significantly altered in the PD group.

	log_2FC	$-log_{10}(P_{adj})$
Cer 16:1;O2/16:0	-0.233838	3.290438
Cer 16:1;O2/18:0	-0.323914	2.727835
Cer 16:1;O2/20:0	-0.195197	3.267713
Cer 16:1;O2/22:0	-0.246216	3.586199
Cer 16:1;O2/23:0	-0.261845	3.658379
Cer 16:1;O2/24:0	-0.253948	2.151607
Cer 16:1;O2/25:0	-0.161415	2.151607
Cer 17:1;O2/22:0	-0.138407	2.045419
Cer 17:1;O2/23:0	-0.139563	8.809093
Cer 17:1;O2/24:1	-0.172939	9.546182
Cer 17:1;O2/25:1	-0.141765	7.162382
Cer 18:0;O2/22:0	-0.358137	2.959724
Cer 18:0;O2/23:0	-0.352533	2.847103
Cer 18:0;O2/24:0	-0.293674	2.012578
Cer 18:0;O2/24:1	-0.223256	2.278197

Cer 18:0;O3/22:0	-0.192654	5.121596
Cer 18:1;O/25:0;O	-0.350337	5.121596
Cer 18:1;O2/22:0;O	-0.326856	2.297416
Cer 18:1;O2/24:0;O	-0.234724	2.152389
Cer 18:2;O2/23:0	-0.147303	2.047969
Cer 18:2;O2/24:0	-0.141776	4.391873
Cer 19:1;O2/22:0	-0.365984	5.07762
Cer 19:1;O2/23:0	-0.351497	5.121596
Cer 19:1;O2/24:0	-0.336938	3.290438
GM3 16:1;O2/22:0	-0.226474	3.280219
GM3 18:1;O2/22:0;O	-0.181719	2.875356
GM3 18:1;O2/24:1	0.180452	3.280219
GM3 18:1;O2/24:2	0.200363	3.658379
GM3 18:2;O2/24:1	0.194616	3.280219
Hex2Cer 18:2;O2/24:0	-0.137995	2.169465084
Hex3Cer 18:1;O2/24:0	0.172383	2.219052
HexCer 18:1;O2/22:0;O	-0.185872	2.848746

HexCer 18:1;O2/24:1	0.187006	3.032166
HexCer 18:1;O2/24:2	0.152857	2.05227
HexCer 18:1;O2/25:0	0.188035	2.278197
HexCer 18:2;O2/24:1	0.190461	2.959724

log_2FC, log_2-fold change

Table S3. Associations between sphingolipids and disease severity assessed by multivariate linear regression.

Lipid species	MDS-UPDRS part III				MMSE			
	Estimate	95% CI		P_{adj}	Estimate	95% CI		P_{adj}
Cer 16:1;O2/16:0	-1.78	-7.72	4.16	0.87	0.51	-1.09	2.11	0.98
Cer 16:1;O2/18:0	-1.82	-6.19	2.55	0.72	0.34	-0.84	1.52	0.98
Cer 16:1;O2/20:0	-0.78	-5.45	3.90	0.97	0.41	-0.85	1.67	0.98
Cer 16:1;O2/22:0	-0.43	-5.18	4.31	0.98	0.45	-0.83	1.72	0.98
Cer 16:1;O2/23:0	0.89	-3.46	5.25	0.96	0.05	-1.13	1.22	0.98
Cer 16:1;O2/24:0	-1.32	-6.10	3.46	0.88	0.66	-0.63	1.94	0.98
Cer 16:1;O2/24:1	1.34	-3.75	6.42	0.88	0.04	-1.33	1.41	0.98
Cer 16:1;O2/25:0	0.73	-3.67	5.13	0.97	0.12	-1.07	1.30	0.98
Cer 17:1;O2/16:0	1.09	-6.31	8.50	0.98	0.43	-1.56	2.43	0.98
Cer 17:1;O2/22:0	0.85	-4.22	5.93	0.97	0.08	-1.29	1.45	0.98
Cer 17:1;O2/23:0	2.16	-2.52	6.85	0.70	-0.15	-1.41	1.11	0.98
Cer 17:1;O2/23:1	-5.14	-10.45	0.17	0.33	0.95	-0.49	2.38	0.98
Cer 17:1;O2/24:0	-0.06	-5.32	5.20	0.99	0.41	-1.01	1.82	0.98

Cer 17:1;O2/24:1	-2.76	-8.00	2.49	0.65	0.49	-0.92	1.91	0.98
Cer 17:1;O2/25:1	-7.11	-12.59	-1.63	0.25	1.41	-0.07	2.90	0.98
Cer 17:1;O2/25:2	-0.89	-6.56	4.78	0.97	0.24	-1.29	1.77	0.98
Cer 18:0;O2/22:0	1.41	-3.09	5.91	0.87	-0.91	-2.11	0.30	0.98
Cer 18:0;O2/23:0	2.18	-2.14	6.50	0.65	-0.96	-2.12	0.20	0.98
Cer 18:0;O2/24:0	1.31	-3.58	6.20	0.88	-0.64	-1.95	0.67	0.98
Cer 18:0;O2/24:1	3.56	-1.37	8.50	0.52	-0.82	-2.15	0.51	0.98
Cer 18:0;O3/22:0	0.70	-4.49	5.89	0.98	-0.28	-1.68	1.12	0.98
Cer 18:0;O3/23:0	2.54	-2.27	7.35	0.65	-0.62	-1.92	0.67	0.98
Cer 18:0;O3/24:0	-0.64	-6.07	4.79	0.98	-0.06	-1.52	1.40	0.98
Cer 18:0;O3/24:1	4.19	-1.69	10.07	0.52	-1.06	-2.64	0.53	0.98
Cer 18:1;O/20:0	-0.17	-4.31	3.98	0.98	0.10	-1.01	1.22	0.98
Cer 18:1;O/21:0	1.09	-2.79	4.98	0.88	-0.31	-1.36	0.73	0.98
Cer 18:1;O/22:0	-2.00	-6.57	2.57	0.71	0.43	-0.80	1.66	0.98
Cer 18:1;O/23:0	-0.85	-5.60	3.89	0.97	0.20	-1.08	1.48	0.98
Cer 18:1;O/24:0	-2.31	-7.47	2.85	0.71	0.60	-0.78	1.99	0.98
Cer 18:1;O/24:1	2.13	-2.84	7.11	0.72	-0.34	-1.68	1.00	0.98
Cer 18:1;O/25:0	0.04	-4.35	4.42	0.99	0.18	-1.00	1.36	0.98

Cer 18:1;O/25:0;O	-0.19	-3.92	3.53	0.98	-0.23	-1.23	0.78	0.98
Cer 18:1;O2/16:0	-0.85	-9.19	7.49	0.98	-0.31	-2.56	1.93	0.98
Cer 18:1;O2/18:0	0.23	-4.62	5.09	0.98	-0.01	-1.32	1.29	0.98
Cer 18:1;O2/20:0	1.30	-4.20	6.79	0.91	-0.04	-1.52	1.44	0.98
Cer 18:1;O2/22:0	0.53	-5.17	6.23	0.98	-0.18	-1.72	1.35	0.98
Cer 18:1;O2/22:0;O	2.03	-1.53	5.59	0.64	-0.09	-1.05	0.88	0.98
Cer 18:1;O2/22:1	2.30	-2.16	6.76	0.65	-0.64	-1.84	0.56	0.98
Cer 18:1;O2/23:0	2.79	-2.62	8.21	0.65	-0.39	-1.85	1.06	0.98
Cer 18:1;O2/24:0	1.74	-3.84	7.31	0.87	0.02	-1.48	1.52	0.98
Cer 18:1;O2/24:0;O	2.93	-0.36	6.21	0.39	-0.07	-0.97	0.82	0.98
Cer 18:1;O2/24:1	3.37	-2.82	9.55	0.65	-0.50	-2.16	1.17	0.98
Cer 18:1;O2/24:1;O	3.19	0.12	6.26	0.33	-0.27	-1.10	0.56	0.98
Cer 18:1;O2/25:0	3.51	-1.61	8.63	0.52	-0.52	-1.91	0.86	0.98
Cer 18:1;O2/25:1	5.17	-0.04	10.39	0.33	-0.56	-1.97	0.86	0.98
Cer 18:1;O2/26:0	4.06	-1.33	9.44	0.52	-0.65	-2.11	0.80	0.98
Cer 18:1;O2/26:1	3.63	-0.68	7.93	0.43	-0.62	-1.78	0.54	0.98
Cer 18:2;O2/16:0	-4.49	-12.15	3.17	0.62	0.99	-1.08	3.05	0.98
Cer 18:2;O2/18:0	-3.74	-8.65	1.17	0.52	0.45	-0.88	1.77	0.98

Cer 18:2;O2/20:0	-3.33	-8.49	1.83	0.53	0.74	-0.65	2.13	0.98
Cer 18:2;O2/22:0	-5.23	-10.74	0.27	0.34	0.99	-0.50	2.48	0.98
Cer 18:2;O2/23:0	-3.66	-9.14	1.82	0.52	0.69	-0.79	2.17	0.98
Cer 18:2;O2/24:0	-6.94	-12.47	-1.41	0.25	1.40	-0.09	2.90	0.98
Cer 18:2;O2/24:1	-0.80	-6.68	5.08	0.98	0.23	-1.35	1.81	0.98
Cer 18:2;O2/25:0	-3.78	-8.87	1.31	0.52	0.61	-0.76	1.99	0.98
Cer 18:2;O2/26:0	-2.79	-8.05	2.48	0.65	0.16	-1.26	1.58	0.98
Cer 19:1;O2/22:0	-0.10	-3.83	3.63	0.98	-0.29	-1.30	0.71	0.98
Cer 19:1;O2/23:0	0.68	-2.85	4.21	0.96	-0.41	-1.36	0.53	0.98
Cer 19:1;O2/24:0	-0.18	-3.99	3.63	0.98	-0.23	-1.26	0.80	0.98
Cer 19:1;O2/24:1	1.10	-2.88	5.08	0.88	-0.44	-1.51	0.63	0.98
GM3 16:1;O2/20:0	-1.59	-6.77	3.59	0.87	0.97	-0.42	2.36	0.98
GM3 16:1;O2/22:0	-2.31	-7.77	3.15	0.72	1.19	-0.27	2.65	0.98
GM3 18:1;O2/16:0	-1.11	-10.40	8.18	0.98	0.83	-1.67	3.33	0.98
GM3 18:1;O2/18:0	-4.87	-11.77	2.04	0.52	1.31	-0.55	3.17	0.98
GM3 18:1;O2/20:0	-0.57	-7.24	6.10	0.98	0.65	-1.15	2.44	0.98
GM3 18:1;O2/22:0	-0.22	-7.61	7.18	0.98	0.21	-1.78	2.20	0.98
GM3 18:1;O2/22:0;O	4.06	-0.05	8.17	0.33	-0.34	-1.45	0.78	0.98

GM3 18:1;O2/23:0	3.60	-2.89	10.09	0.65	-0.09	-1.84	1.66	0.98
GM3 18:1;O2/24:0	4.22	-2.06	10.51	0.52	-0.09	-1.79	1.60	0.98
GM3 18:1;O2/24:0;O	5.88	2.17	9.59	0.21	-0.45	-1.47	0.56	0.98
GM3 18:1;O2/24:1	2.34	-4.51	9.20	0.83	0.12	-1.73	1.97	0.98
GM3 18:1;O2/24:1;O	5.34	0.63	10.05	0.30	-0.27	-1.55	1.01	0.98
GM3 18:1;O2/24:2	2.63	-4.06	9.31	0.76	-0.01	-1.82	1.79	0.98
GM3 18:2;O2/16:0	-6.15	-14.97	2.67	0.52	1.63	-0.74	4.01	0.98
GM3 18:2;O2/18:0	-7.30	-13.91	-0.69	0.30	1.86	0.07	3.64	0.98
GM3 18:2;O2/22:0	-6.94	-13.70	-0.17	0.33	1.82	0.00	3.64	0.98
GM3 18:2;O2/24:0	-6.02	-12.06	0.02	0.33	1.51	-0.12	3.13	0.98
GM3 18:2;O2/24:1	0.60	-5.82	7.02	0.98	0.66	-1.07	2.39	0.98
Hex2Cer 16:1;O2/16:0	-4.37	-10.17	1.43	0.52	1.32	-0.24	2.88	0.98
Hex2Cer 18:1;O2/14:0	2.63	-2.89	8.15	0.68	-0.05	-1.54	1.44	0.98
Hex2Cer 18:1;O2/16:0	0.80	-7.44	9.04	0.98	0.65	-1.57	2.87	0.98
Hex2Cer 18:1;O2/22:0	3.87	-2.46	10.19	0.58	0.18	-1.53	1.88	0.98
Hex2Cer 18:1;O2/23:0	3.35	-1.72	8.41	0.53	0.26	-1.10	1.63	0.98
Hex2Cer 18:1;O2/24:0	6.76	0.95	12.57	0.30	-0.34	-1.92	1.24	0.98
Hex2Cer 18:1;O2/24:1	2.81	-3.58	9.20	0.71	0.24	-1.48	1.96	0.98

Hex2Cer 18:1;O2/24:2	0.66	-4.87	6.20	0.98	0.68	-0.81	2.16	0.98
Hex2Cer 18:2;O2/16:0	-5.29	-12.68	2.09	0.52	1.52	-0.47	3.51	0.98
Hex2Cer 18:2;O2/24:0	-1.33	-8.18	5.52	0.96	1.43	-0.40	3.27	0.98
Hex2Cer 18:2;O2/24:1	-0.37	-6.27	5.53	0.98	0.80	-0.78	2.39	0.98
Hex3Cer 18:1;O2/16:0	-0.88	-3.47	1.70	0.83	0.10	-0.60	0.79	0.98
Hex3Cer 18:1;O2/18:0	2.62	-4.23	9.46	0.77	0.71	-1.13	2.55	0.98
Hex3Cer 18:1;O2/22:0	5.63	-0.01	11.26	0.33	-0.26	-1.79	1.27	0.98
Hex3Cer 18:1;O2/23:0	5.97	1.64	10.30	0.21	-0.46	-1.64	0.73	0.98
Hex3Cer 18:1;O2/24:0	6.48	1.96	10.99	0.21	-0.38	-1.62	0.85	0.98
Hex3Cer 18:1;O2/24:1	5.42	-0.74	11.59	0.39	-0.10	-1.77	1.57	0.98
Hex3Cer 18:2;O2/24:0	0.32	-6.01	6.66	0.98	0.71	-0.99	2.42	0.98
Hex3Cer 18:2;O2/24:1	3.27	-3.20	9.74	0.65	0.11	-1.64	1.85	0.98
HexCer 17:1;O2/24:0	1.29	-3.98	6.56	0.90	0.32	-1.09	1.74	0.98
HexCer 18:1;O2/16:0	1.19	-6.37	8.75	0.97	0.18	-1.85	2.22	0.98
HexCer 18:1;O2/16:0;O	5.64	-2.01	13.30	0.52	-0.07	-2.14	2.00	0.98
HexCer 18:1;O2/18:0	0.21	-6.06	6.49	0.98	0.21	-1.48	1.90	0.98
HexCer 18:1;O2/20:0	-0.20	-6.30	5.90	0.98	0.56	-1.08	2.21	0.98
HexCer 18:1;O2/22:0	0.10	-5.91	6.10	0.99	0.41	-1.20	2.03	0.98

HexCer 18:1;O2/22:0;O	4.46	0.13	8.80	0.33	-0.61	-1.78	0.56	0.98
HexCer 18:1;O2/23:0	2.97	-2.60	8.55	0.65	-0.11	-1.61	1.39	0.98
HexCer 18:1;O2/23:0;O	4.79	0.95	8.62	0.25	-0.51	-1.55	0.53	0.98
HexCer 18:1;O2/24:0	3.76	-2.00	9.52	0.53	-0.07	-1.62	1.49	0.98
HexCer 18:1;O2/24:0;O	5.09	1.39	8.79	0.21	-0.41	-1.42	0.60	0.98
HexCer 18:1;O2/24:1	4.27	-1.91	10.46	0.52	-0.26	-1.93	1.41	0.98
HexCer 18:1;O2/24:1;O	4.60	0.54	8.67	0.30	-0.46	-1.57	0.64	0.98
HexCer 18:1;O2/24:2	5.16	-0.71	11.04	0.39	-0.09	-1.68	1.50	0.98
HexCer 18:1;O2/25:0	3.87	-1.50	9.23	0.52	-0.18	-1.63	1.27	0.98
HexCer 18:2;O2/22:0	-6.10	-12.39	0.18	0.33	1.59	-0.11	3.28	0.98
HexCer 18:2;O2/23:0	-4.15	-10.19	1.89	0.52	1.17	-0.46	2.80	0.98
HexCer 18:2;O2/24:0	-5.88	-12.42	0.66	0.39	1.66	-0.10	3.42	0.98
HexCer 18:2;O2/24:1	1.64	-4.39	7.66	0.88	0.25	-1.38	1.87	0.98
HexNAcHex3Cer 18:1;O2/16:0	0.33	-8.70	9.36	0.98	0.79	-1.64	3.22	0.98
HexNAcHex3Cer 18:1;O2/24:1	8.07	0.90	15.23	0.30	-0.29	-2.24	1.66	0.98

HexNAcHex3Cer 18:2;O2/16:0	-3.97	-11.98	4.05	0.66	1.47	-0.68	3.63	0.98

Models are adjusted for age, sex, disease duration, and levodopa equivalent daily dose.

Reference

1. Schmid R, Heuckeroth S, Korf A, Smirnov A, Myers O, Dyrlund TS, et al. Integrative analysis of multimodal mass spectrometry data in MZmine 3. Nat Biotechnol. 2023;41(4):447–9.

2. Peng YF. SphingolipidsID.jl (Version 0.3.0). 0.3.0 ed. https://github.com/yufongpeng/SphingolipidsID.jl 2024.

3. Tsugawa H, Ikeda K, Tanaka W, Senoo Y, Arita M, Arita M. Comprehensive identification of sphingolipid species by in silico retention time and tandem mass spectral library. J Cheminformatics. 2017;9.
